# Sweden’s engagement in global health: a historical review

**DOI:** 10.1186/s12992-019-0499-1

**Published:** 2019-11-26

**Authors:** Rachel Irwin

**Affiliations:** Department of Arts and Cultural Sciences, Lund University, Lund, Sweden

**Keywords:** Sweden, Global health, History, Diplomacy, Welfare, development

## Abstract

**Background:**

Sweden is a long-standing and significant contributor to overseas development aid. This commitment to global health and development is part of *Sverigebilden*, or the view of Sweden in the world that is formally promoted by the Swedish government. Sweden is seen by many in the global health community as leader on human rights and health and has traditionally been one of the most engaged countries in multilateral affairs more broadly.

**Results:**

This article places Sweden’s engagement in global health within the wider context of domestic changes, as well as transitions within the broader global health landscape in the post-World War Two (WWII)- era. In doing so, it reviews the globalization of health from a Swedish perspective. It also addresses broader questions about what it means for a country to be ‘active’ or ‘engaged’ in global health and responds to recent suggestions that Swedish influence in health has waned. The article finds that in Sweden there is wide political consensus that international development and global health engagement are important, and both are part of the maintenance of *Sverigebilen.* While there is a not one single Swedish approach to global health, there are norms and values that underpin global health engagement such as human rights, solidarity, equity and gender equality. A sustained focus on key issues, such as sexual and reproductive rights and health (SRHR), creates a tradition which feeds back into *Sverigebilden.*

**Conclusions:**

The Swedish experience demonstrates the linkages between foreign and domestic policies with regard to international health and development, and to the globalization of public health practice and diplomacy. In global health *Sverigebilden* is tied to credibility. Sweden is able to exercise influence because of a successful welfare model and strong research traditions; conversely, long-standing and new threats to this credibility and to *Sverigebilden* pose challenges to Sweden’s future engagement in global health.

## Background

Sweden is a long-standing and significant contributor to overseas development aid, consistently in the top 10 donor countries in total spending and at the top of the list when measured as a percentage of Gross National Income (GNI) [1, 2]. When measured as a percentage of GNI, Sweden has been above the Donor Assistance Committee (DAC) average since 1969 and was the first donor country to meet the 0.7% target (in 1975, along with the Netherlands) [3]. Looking specifically at health-related aid, Sweden also stands out: extrabudgetary funding by Sweden to the World Health Organization (WHO) from 1948 up until 1991 surpassed that of the United States [4], and while the US has overtaken Sweden in total amount, Sweden remains the top donor per capita. Sweden was one of the six original donors to Gavi, the Vaccine Alliance and has “steadily” increased its “political and financial commitment [5].” It is also the eight largest government donor to the Global Fund to Fight AIDS, Tuberculosis and Malaria (Global Fund), behind much larger countries such as the US, Canada and Germany [6]. Public opinion on aid is high, with 96% of Swedes believing that ‘it is important to help people in developing counties.’ [7] Beyond traditional development assistance for health, the government of Sweden, supported by research and civil society, has been active in pushing for solutions to global health challenges, such as sexual and reproductive health and rights and antimicrobial resistance.

This commitment to global health and development is part of *Sverigebilden*, or the view of Sweden in the world that is formally promoted by the Swedish government [8, 9]. Sweden is seen by many in the global health community as leader on human rights and health, and has traditionally been one of the most engaged countries in multilateral affairs more broadly [10]. Yet, little over 100 years ago Sweden was one of the poorest countries in Europe. It was a highly unequal society and was not even a full democracy until 1921. Since then it has developed into a welfare state which includes a health system based on the principles of solidarity, equity and cost-effectiveness and consistency ranks high on measures of quality of life.

Building upon the existing literature on Swedish foreign aid and Swedish public health [11–13], this is the first study to look at the interplay between the two. That is, this article aims to place Sweden’s engagement in global health within the wider context of domestic changes, as well as transitions within the broader global health landscape in the post-WWII era (See Table 1). In doing so, it reviews the globalization of health from a Swedish perspective. As Sweden is a significant and influential donor, most individuals working in global health interact with Swedish researchers, civil servants and diplomats in international affairs, or are impacted by decisions taken by the government regarding global health engagement. Despite this, the country is typically relegated to a footnote or minor comments in international scholarship on the history of global health. This article aims to fill that gap and provides a guide for understanding Swedish approaches to global health. It also addresses broader questions about what it means for a country to be ‘active’ or to be ‘a leader’ in global health [14], and responds to recent suggestions that Swedish influence in health has waned [15].

**Table 1:** Swedish milestones in domestic and global health

1947. Sweden becomes a party to the WHO’s constitution	
1952. Central Committee for Swedish Technical Assistance to Less Developed Areas (CK) set up	
1955. Alcohol monopoly Systembolaget founded	
1955. Universal health insurance introduced in Sweden	
1956. Introduction of compulsory sexuality education in schools	
1958. Sweden is the first country to provide development assistance for family planning (to Ceylon)	
1961. CK is disbanded and replaced with the Agency for International Assistance (NIB)	
1962. First bill on international development, Government Bill 1962:100, is introduced	
1965. Swedish International Development Authority (SIDA) founded through a reorganisation of NIB.	
1972. Gender equality is made a formal part of government policy	
1974. Abortion legal at the woman’s request up to the 18th week. Abortion was made legal in limited circumstances in 1938.	
1975. Sweden is the first country to meet the 0.7% aid target.	
1975. Swedish Agency for Research Cooperation with Developing Countries (SAREC) is founded.	
1978. Uppsala Drug Monitoring Centre established	
1985. The Conference of Experts on the Rational Use of Drugs in Nairobi is convened, pushed in part by Norway and Sweden.	
1988. 4th International AIDS Conference held in Stockholm	
1995. Re-organisation of Sweden’s international development work into Sida (Swedish International Development Cooperation Agency)	
1995. Sweden joins the EU.	
2000. GAVI founded, Sweden is one of the six original donors.	
2002. The Policy for Global Development sets out that all government decisions should take into account poverty and equity	
2009. Sweden’s presidency of the Council of EU highlights AMR and the need for incentives to develop new antibiotics.	
2014. Feminist Foreign Policy launched.	
2015. Sustainable Development Goals adopted. The thematic consultation on health had been co-organised by the Government of Sweden.	

The article finds that in Sweden there is wide political consensus that international development and global health engagement are important, and both are part of the maintenance of *Sverigebilen.* While there is a not one single Swedish approach to global health, there are norms and values that underpin global health engagement such as human rights, solidarity, equity and gender equality. A sustained focus on key issues, such as sexual and reproductive rights and health (SRHR), creates a tradition which feeds back into *Sverigebilden*. For instance, according to the Feminist Foreign Policy handbook published by the Swedish Ministry for Foreign Affairs in 2018: ‘A part of foreign policy is about promoting Swedish values and spreading *Sverigebilden* around the world. In this picture, gender equality has a clear place.’ [16].

Additionally, a country brings its own domestic experiences, both positive and negative, to its global health engagement [14, 17]. For instance, recent research on Canada and Germany has examined the historical context of domestic priorities, challenges and politics, as well as the role of research in a country’s global health engagement [14, 18]. In the Swedish example, engagement in global health is partly based on a dominant narrative of Sweden’s rapid rise from poverty [19], and on the tackling of specific challenges, such as alcohol misuse or maternal and childhood mortality in the 19th and early 20th Centuries. A caveat is that these narratives present an idealized picture of societal transformation and not all members of society have shared equally in the Swedish model. Yet these narratives, however incomplete, are worth exploring because they have informed global health practice throughout the 20th and into the 21st Centuries. Moreover, Sweden’s engagement in global health is predicated on the belief that research can be used to solve social ills, and – at the best of times – close ties between research, civil society and policy.

A second caveat is that within these norms, values and traditions, there is considerable room for disagreement on the details of policy and implementation. For example, toward the end of the Smallpox Eradication Initiative, the Swedish development agency (Sida) and the the Ministry for Foreign Affairs (MFA) disagreed on donating funding, with the MFA arguing that it was a vertical programme not tied to health systems and Sida believing it was a good cause in spite of this; in fact, waning and waxing tensions between Sida and the MFA have existed since the 1960s over the sharing of responsibilities for setting and implementing aid policy-setting [20, 21]. In the 1980s, political parties argued over whether or not to commit to a 1% target, rather than the 0.7% target [22]. More recently NGOs have protested Sweden’s funding cuts to the Global Fund in 2016 [23] and have called attention to Sweden’s role in diluting language in a World Health Assembly Resolution regarding transparency in pharmaceutical pricing [24]. In spite of disagreements, the starting point for Swedish engagement in global health is that it is a positive endeavour. At the same time, in global health *Sverigebilden* is tied to credibility. Sweden is able to exercise influence because of a successful welfare model and strong research traditions; conversely, threats to this credibility and to *Sverigebilden* pose threats to Sweden’s future engagement in global health.

## Methods and scope

The history of Sweden’s engagement in global heath is reconstructed through secondary materials, including government publications, the official records of both WHO and UN General Assembly meetings, news media, articles in Swedish public health journals and memoirs of key figures. Secondary materials were identified through searches in the National Library of Sweden’s catalogue, GoogleScholar, the Retriever Media Database, the Government of Sweden’s website, the Swedish Parliament’s electronic records, the WHO’s IRIS database, and the UN’s Dag Hammarskjöld’s library catalogue. Search terms were related to international health, global heath, the World Health Organization and development aid, and searches were carried out in both English and Swedish. The article further draws upon materials from the Swedish National Archives (Riksarkivet) and the World Health Organization’s archives in Geneva, including correspondence and records of meetings. Although not all sources reviewed are cited due to space constraints, the source material was used to identify additional material or to verify names and dates.

Interviews were conducted with 16 individuals during the period 2017–2018. Eight interviewees were senior figures in Swedish global health and/or development, six of whom were retired or semi-retired. A further eight were civil servants or diplomats currently working in global health. The interviewees represented experiences from the main governmental actors involved in global health: the National Board of Health and Welfare (Socialstyrelsen, previously Medicinstyrelsen), Ministry of Health and Social Affairs (Socialdepartementet), Ministry for Foreign Affairs (Utrikesdepartementet), Sida (Styrelsen för internationellt utvecklingssamarbete) and the Public Health Agency (Folkhälsomyndigheten). The individuals also had experience working within international organizations (including the WHO), other governmental organisations, civil society and the research community.

The interviews were semi-structured. The interviews with senior figures took the form of oral history and were focussed on the individual’s experiences and views on how global health had changed over time. The interviews with current civil servants and diplomats focussed on their everyday working lives, the relationships between their agency/department and the WHO, and Swedish priorities in health. Although very little of the interview material is formally presented here, it was used to fill gaps in the documentary record, to identify further individuals and incidents for follow-up and to gain a broader context on Sweden’s engagement for use in analysing the documentary record. Both the interview material and documentary record were analysed using approaches from grounded theory [25]. That is, themes and questions arose during the course of the research.

While recognising that the changes in terminology – from hygiene and tropical medicine to global health – have material consequences, for the sake of simplicity the terms international and global health are used somewhat interchangeably throughout the article [26, 27]. Global health itself is a poorly defined concept [27, 28]. On one hand, it can be defined as ‘those issues that transcend national boundaries and governments and call for actions on the global forces that determine the health of people.’ [29] However, in practice it is often used interchangeably with development aid or, at the very least it is associated with improving health in low and middle-income countries. This article aims at addressing the first definition whilst recognising much of the research material is focussed on the latter. Moreover, examining all aspects of Sweden’s engagement with global health is outside of the scope of any single article and practically a focus on international development and the cooperation with international organisations is a simpler task than examining all aspects of global health [14]. Similarly, the article’s focus is on the government’s engagement in global health, although this is discussed in relation to the research community and civil society. The article provides an overview, with the caveat that any decade or episode in Sweden’s global health history warrants its own investigation. It also largely ignores both humanitarian aid and regional health engagement, such as that within the WHO European Region or the health-related European Union agencies; the important role of Nordic cooperation in global health is also largely outside the scope of this article.

In the sections that follow, the article presents six overlapping eras in global health and the interplay describing between Swedish and international actors within the context of economic, political and social changes, both domestically and internationally. The final section concludes by examining the norms, values and traditions that characterise Sweden’s engagement in global health and by reflecting on its future role in the sector.

## Results

### The pre-history of Sweden’s engagement in global health

In the 1800s and early 1900s, many Swedes lived in the context of poverty and inequality. An increase in population coupled with a shortage of quality farmland led to a situation of downward social mobility: typically only one child could inherit land and the rest were forced into various types of insecure wage-labour or even forced labour [11]. Northern Sweden suffered from a famine so severe (1867–1869), that aid was sent to Sweden from abroad [30]. The country also experienced the challenges associated with early industrialisation such as mass urbanisation, worker safety, and poor housing conditions. The conditions at home pushed nearly a quarter of the country’s population to emigrate between 1840 and 1920, mainly to North America.

Concerned by this mass emigration, the parliament called for a commission to investigate the underlying causes [31]. The findings, which focused on issues of class inequality, economic opportunities, and better living conditions, was one contributing factor to the formation of the modern Swedish welfare state. There was also an early focus on research and statistics. An agency for the collection of public records (Tabellverket) was established in 1749 and later was replaced by Statistics Sweden in 1860, while the National Medical Board (Medicinstyrelsen) was established as the authority over the medical profession, including hospitals, in 1877, replacing earlier institutions dating back to 1663 [11]. It was through this expertise in both data collection and the bureaucratisation of medicine that Sweden was able to contribute to early international health cooperation, for instance through active membership in the Office International d’Hygiéne Publique and the Health Organization of the League of Nations [32, 33].

Although the collective memory of the poverty and inequality of that period in Swedish history has faded, it was very much in the minds of those active in early international health efforts. Several aspects of societal transformation stand out during this period with relevance for later health engagement [11].

Firstly, there was an early concern with reproductive health. This was found in unique efforts to reduce maternal mortality, starting as early as the 1600s; these reductions were due in large part to the introduction of midwifery [34]. A key example was the campaigning of Elise Ottesen-Jensen, who pushed for sexuality education and rights, founding the Swedish Association for Sexuality Education (RFSU) in 1933. Fears over a declining population led to the creation of a Royal Commission in 1935 which set out that the role of the state is to create an environment which would support people to have children. It also saw family planning as a way to decrease child poverty: the idea that every child should be a wanted child [35, 36]. As part of these movements, Sweden was also early to legalise abortion (in certain circumstances), and access to information about contraceptives.

High alcohol consumption and related harms, along with the fact that many parliamentarians and influential individuals in international health were part of international temperance movements, or at least teetotallers themselves, gave rise to alcohol control efforts which still exist in the form of Systembolaget, the state-run alcohol retail monopoly [11, 37]. More broadly, this contributed to the normalisation of the idea that industry can be controlled, a notion that is part of the ‘Swedish Middle Way.’ The Swedish Middle Way was a concept introduced by American journalist Marquis Childs in 1936, who described the Swedish political, economic and social systems as a compromised between capitalism and communism. While somewhat of a simplification, the term has been used as part of *Sverigebilden*: that is, the Swedish system is supposedly based on a strong capitalist system which generates wealth but uses this wealth to temper the worst aspects of capitalism.

Both the alcohol issue and reproductive health demonstrate an approach to policy-making based on rationality and research. That is, the response to early health issues, and the founding of the welfare state more broadly, was characterised by a shift in seeing social ills such as alcoholism or poverty as problems to be solved, rather than as individuals’ moral failings. These responses also set out the belief that a society can be planned and created. For instance, politician and diplomat Alva Myrdal used research on children and the family to call for gender-related legislation, such as facilitating married women’s entry into the workforce. However, this underlying belief that the state should intervene in the health of individuals for the common good was used to justify coerced and forced sterilisation, along with abortions for “eugenic reasons” and unethical dental experiments on patients at Vipeholm Hosptial (1945–1955) [38, 39].

### 1945- early 1960s: early internationalism

After the end of the Second World War, Sweden resumed welfare state-building and saw the roll-out of various programmes, including universal health insurance (1955) and compulsory sexuality education in schools (1956). This expansion of the welfare state was supported by a strong economy and the subsequent tax base to pay for services. Within this context, Swedish development policy was an extension of welfare state-building, not least because people who were active in global health were also active in welfare state-building at home [39]. In one example Axel Höjer, head of Medicinstyrelsen (1935–1952), later worked in India and Ghana (1952–1960) for the WHO [19, 39–41].

As a neutral country during the war and a non-aligned one during peace time, the Swedish government also saw itself as being in strong position to contribute to the international spirit of peacebuilding and cooperation. There were initial concerns over joining the UN – both from Swedes concerned with joining a security organisation and from countries unhappy with how Sweden’s neutrality had benefitted both sides of the war. However, the idea of the WHO and Sweden’s participation within it, was met with enthusiasm and Sweden became a party to the WHO’s Constitution on 28 August 1947 [19, 33, 37, 42].

Global health, via the WHO, was and largely (but not solely) focussed on infectious disease, with issues including polio, standardization of pharmaceutical products, and problems with health worker training, malaria, maternal and child health, hygiene and clean water, healthcare/administration, cholera, venereal disease tuberculosis, typhoid and cancer comprising the bulk of the WHO’s work [32, 33, 43]. While international health engagement addressed health concerns faced by many countries, from the Swedish perspective, international health vis-a-vis the WHO was ‘no longer a question of protecting the USA and Europe from dangerous contagious disease’ but also about ‘helping the poor, often oppressed, poorly developed countries in the field of health care [43]. To this engagement, ‘Swedes [were able] to make important contributions to the WHO’s work through their specialist knowledge, by assisting with the special views and experiences acquired in Sweden with [their] disease panorama and [their] research work as a background.’ [44]

Although international health cooperation in the post-war era was largely dominated by the WHO, Swedes participated in or cooperated with other non-governmental organisations and multilateral bodies, such as UNICEF, NGOs, such as Save the Children and the Red Cross Movement [43, 45]. To support this international work, the Ministry for Foreign Affairs set up Central Committee for Swedish Technical Assistance to Less Developed Areas, or CK, in 1952. CK was a grouping of associations and popular movements, including representatives of trade unions, adult education organizations and the temperance movement, missionary societies, and others. While not a colonial power, Sweden had established mission societies in Africa in the 1880s, which included health and educational programmes, and these societies influenced later aid work. For example, Ethiopia was one of Sweden’s first recipient countries post-WWII, in part due to relationships already established by missionary societies [46].

CK served not only to set up Swedish development assistance, but also to promote popular support in Sweden for their work, a task in which they were largely successful [46]. The election of Dag Hammarskjöld to be the 2nd UN Secretary General in 1953 also increased awareness of international affairs and development amongst the general Swedish population [39]. Swedish approaches to development cooperation were formalised in the first government bill on aid in 1962, Government Bill 1962:100, which stressed the moral duty and international solidarity aspects of aid [39, 47].

One of the most contested issues of this era was the population question. After WWII, many countries in the so-called Third World were characterised by a rapid demographic transition: falling mortality rates, but relatively stable birth rates, leading to population growth. At the same time, there was a belief that these countries were not economically developed enough to handle the transition and there was international concern over overpopulation [35]. Based in part on its earlier experiences in reducing maternal mortality and domestic concern over child poverty and de-population, with Sweden, along with Norway, pushed an early maternal and child health (MCH)-oriented agenda [33, 35, 48]. Again some of the individuals involved in international health and foreign policy had had been active in these health issues domestically, such as Alva Myrdal, so there was a strong passion for pushing family planning in international health; this was supported by the relative openness for discussing sexual and reproductive matters in Sweden [49]. In 1958, Sweden was the first country to provide development assistance for family planning, responding to a request from Ceylon and then later to Pakistan in 1961 [50, 51].

However, some countries had moral and religious objections to family planning. Others, including Soviet counties and many in the so-called “Third World” were concerned that population control was being promoted in lieu of development aid. That is, they felt that family planning was being promoted in order to reduce the number of people in the Third World, thus reducing the need for development aid [35, 52]. Additionally, while Sweden’s early involvement in family planning was seen as ‘enlightened’ at the time and also in historical context, part of the motivation came from this belief that state should intervene in the reproductive health of individuals for the common good, a belief that led to forced sterilisations even into the twenty-first Century in Sweden [11, 39, 53].

### 1960s- early 1980s: global health becomes radical

Swedish development aid was informed by domestic and international movements, including the student and women’s movements, and subsequent domestic policy reforms such as the introduction of gender equality as a formal part of government policy, the abolition of joint taxation of spouses (1972), the legalisation of abortion at a women’s request, rather than needing a doctor’s permission (1974), and creation of day care and parental leave (1974). It was a radical time in Swedish society and globally, which impacted upon development aid.

Global health has always been political [54], but towards the end of the 1960s material changes in the international landscape led to substantial reforms. Decolonialisation, starting in the 1950s and moving into the 1960s and 1970s, saw what was then called the Third World’[emerge] from obscurity and in many ways become paramount in WHO, if not in action at least in rhetoric.” [4] Global health became political in part because increasing membership into the UN agencies in the form of independent states brought to light the problems in low and middle-income countries (LMIC) [52, 55].

Sweden had walked a fine line during colonisalisation, on one hand supporting de-colonialisation efforts but also still often siding with colonial powers, who were its main trading partners [41]. Similarly, Sweden’s support for decolonialsation was partly about solidarity, but also served Swedish interests [39]. That is, as a non-aligned country it had an interest in forming strong diplomatic and trade relationships with newly independent states, and aid was seen as an explicit way to promote peace and security. Sweden became increasingly active in development and supporting decolonisation under prime minister Olof Palme (1969–76 and 1982–6) and its role as a non-aligned, non-colonial power gave it more ‘credibility’ with the newly emerging states and it was ‘thus considered “enlightened” by many developing countries.’ [33, 39, 56, 57] This was in contrast to both the Eastern and Western blocks that had ‘employed health efforts as a means of influencing (and politically dominating) underdeveloped countries, including former colonies, as part of the larger Cold War struggle.’ [58]

Domestically, development work was largely the domain of both the Swedish International Development Authority (SIDA) and Swedish Agency for Research Cooperation with Developing Countries (SAREC). CK was disbanded in 1961, with the rationale that a state-run body should be responsible: The Agency for International Assistance, or NIB, was founded in 1961 and later re-organised into SIDA, in 1965, as a way to ‘bureaucratize, organize and structure the development aid.’ [20, 46] It was joined in 1975 by SAREC which was founded with the explicit idea that science and research could solve development challenges.

By the late 1960s there was also a shift of opinion in the ‘population issue,’ in part due to technical feasibility in the form of new contraceptives, but also because of continued concern over rapid population growth [59]. For instance, while the WHO had avoided dealing with ‘social, cultural and economic’ issues because they were not medical and therefore outside of its mandate, there was also a graduate shift here. In 1966 the World Health Assembly (WHA), the WHO’s main governing body, decided it was acceptable for the WHO to give technical advice on family planning ‘on request’ and by the early 1970s the situation had developed to the extent that WHO’s Special Programme of Research, Development and Research Training in Human Reproduction (HRP) was created in 1972, in large part at the behest of Sweden [59]. In the late 1960s Sweden was also the first country to provide support to the International Planned Parenthood Federation (IPPF), founded in part by Elise Ottersen-Jensen in 1953, and to support the United Nations Population Fund (UNFPA) [59]. Finally, there was also a gradual shift from family planning as a tool to address the ‘population issue’ to one of development and human rights, which continued on into the 1980s and 1990s.

The election of Halfdan Mahler as Director-General of the WHO also led the push for a more radical international health agenda, based on equity and social justice and the emergence a new health for all paradigm. This is also the era in which the WHO moved from being primarily a medical and normative organisation to including advocacy as part of its remit [52, 55]. For instance, the late 1970s saw the adoption of WHA30.43 on Health for All, the Alma-Ata Conference on Primary Health Care, and the Essential Medicines List (1977) and the 1979 Primary Health Care Strategy (1979).

Both the Swedish government and civil society were supportive of this work, although there were reservations that international action would not go far enough. For example, during this time, the Dag Hammarskjöld Foundation held seminars which contributed to the conversations around Alma Ata (1977) and to work around access to essential medicines in order to push the government into stronger action [60, 61]. Later, in 1985, at the request of WHA 37.33, or the so-called “Nordic Resolution,” put forward by Sweden, the WHO convened the Conference of Experts on the Rational Use of Drugs in Nairobi in order to ‘to discuss the means and methods of ensuring the rational use of drugs, in particular through improved knowledge and flow of information, and to discuss the role of marketing practices in this respect, especially in developing countries.’ [62, 63] The lead up to the conference was characterised by Mahler as being ‘surrounded’ by ‘a year and a half of unprecedented social pathology’ [64] and, in fact, representatives of the US pharmaceutical industry pressured the US State Department and other governments in the run-up to it. In the end, however, the meeting was seen as largely successful, characterising a strong WHO and partnership in the form of Swedish and Nordic engagement.

The radical spirit characterising the WHO’s work continued into the drafting and adoption of the UNICEF/WHO Code of Marketing of Breastmilk Substitutes. This was a watershed moment, in which NGOs increasingly became involved in advocacy at the WHO and also further set the precedence for the WHO to regulate business interests. Sweden was one of the countries that put it on the agenda and went on to support NGOs working on the Code [20, 65]. Swedish research significantly contributed to the development of the Code, with projects led by Yngve Hofvander and Bo Wickström informing the process. Göran Sterky, then head of the WHO’s Department of Maternal and Child Health played a ‘pivotal role’ the development of the Code, even earning ‘himself the honour of being the top name on a blacklist industry had made of WHO staff members “not to be trusted.” [66]

Domestically, the Code largely had domestic support in part because the infant formula industry was also qualitatively different than in other countries. Findus (then owned by Nestlé) and Semper had had a voluntary agreement since 1964 to not advertise infant formula to mothers until a child was 3 months old [67]. Semper was largely reliant on the domestic market, rather than being a major exporter. Domestically, Semper was threatened with a boycott primarily for their involvement with a Turkish producer of infant formula and Findus was boycotted as part of the international Nestlé boycott, organised in Sweden by Konsumentgillesförbundet [68–70]. There was also concern by breastfeeding groups that the Swedish law was not as strong as the Code, which was ‘embarrassing.’ [71] On the other side of the argument, there was criticism that the implementation of the code would be against freedom of expression (Tryckfrithetsförordningen). There were also criticisms from industry that the problems of marketing infant formula were of concern to low-income countries but were not valid in a high-income country like Sweden, although the official Swedish response was that it was an ‘action of solidarity with developing countries,’ again reiterating the solidarity values underpinning Swedish international health engagement [72].

### 1980s–1990s: HIV, neoliberalism and women’s rights

The 1980s was characterised by significant changes in the global health landscape, including the entry of the World Bank, UNICEF, and the European Economic Community (EEC) into policy analysis, health economics, and health planning and management [4].Although support for aid remained high, economic difficulties domestically led, in part, to a shift toward increased concern with the efficiency and effectiveness of aid [56] [21]. These debates had already begun during the global economic crisis of the 1970s but were further given a platform during the Swedish banking crisis of the 1990s and were coupled with wider domestic critiques of the welfare state. Internationally, they were also later tied to conversations such as the 1998 “Assessing Aid” report by the World Bank, which highlighted an emerging aid effectiveness agenda [21, 73].

The aid effectiveness agenda itself was a manifestation of the rise of neoliberalism in international health. Rushton and Williams (2012) have noted the way in which neoliberalism has ‘colonised’ global health by normalising the ‘deployment and privileging of market-based policy responses, to commodification, privatisation, liberalisation of health and healthcare, and to the individualisation of risk and responsibility for health.’ Practically, the emergence of business and finance-oriented actors, such as the World Bank, as international health actors health saw an emphasis on ‘reaching concrete goals through management-style performance accountability measures in place of the preceding era’s broader assessments of health and social wellbeing.” [58] On one hand, by ‘making the case for health to heads of state and finance ministers,’ documents such as the World Development Report 1993: Investing in Health also led to increased attention and financing to global health [74]. However, the dominant analysis within global health scholarship is that neoliberalism been damaging to public health and that neoliberal policies, as promoted by the World Bank, emphasized efficiency at the expense of equity [60, 75].

For example, in a meeting organised by the Dag Hammarskjöld Foundation in 1996, the participants, a mix from the North and South, discussed how the World Bank and regional development banks had distorted markets through structural adjustment policies which in turn had led many low and middle-income countries to cut social services. They also noticed the subsequent privatisation of healthcare which was ‘at odds with the universalist, primary health care-oriented approaches advocated by WHO.’ Concluding that “without equity, promoting health a part of development loses its entire meaning’ delegates felt that:

“there are a wide range of factors that appear to be contributing to this deteriorating world heath situation: a lack of insight into the intersectoral nature of health problems and the failure to act outside the narrowly defined health sector to make health a priority in all sectors of society, distortion of health priorities at national and global levels; a narrow top-down, service-oriented view of health; rapid privatization of healthcare; reduced state support, especially for primary and preventive health care programmes; and tardy implementation of, if not retreat from, the goal of national drug policies. All of these have been aggravated by increasing distortions in the structures of the world economy, under the impact of structural adjustment policies, the persistent indebtedness of the South, and new iniquitous world trade agreements ...

...furthermore, there has been indiscriminate privatization of the health sector with little attempt by the state to regulate it ... many of the ‘reforms’ of the health sector are externally driven ... at their core is a quantitative and narrow notion of health and disability. This notion does not treat health as a human right [60].

The Swedish government was, to a large extent, able to interpret neoliberalism in a way that was consistent with the social democratic values which had dominated politics, in line with the semi-mythical ‘Swedish Middle Way.’ Effectiveness had always been a part of Swedish aid and the Sweden-as-donor interpretation of neoliberalism saw cost-effectiveness as a way to increase equity [21]. At the same time neoliberalism began its dominance, Swedish also stepped up their rights-based and gender-based politics, which may have also tempered the worst aspects of neoliberalism. Moreover, Danielsson and Wohlgemuth (2003) have suggested that

...other donors – including the World Bank – have adjusted their thinking to the Swedish philosophy, by putting an increasing emphasis on non-economic factors and by explicitly recognising that poverty is multi-dimensional and cannot be successfully attacked through economic growth only [76].

Domestically, however, the recession of the 1990s saw the beginnings of cutbacks in the welfare state which would continue into the 2000s and 2010s, ultimately calling into question the credibility of the Swedish model [77].

During this period, HIV/AIDS also emerged as a disrupting force in global health. Domestically, the response was paradoxical. On one hand, the long tradition of sexual education in schools, along with a system of youth clinics and the wider promotion of gender equality, had created a societal openness around sexuality. At the same time the public health response was characterised in part by repressive measures such as closing bathhouses and obligations to disclose one’s HIV status [78–80]. Writing about Sweden’s contribution to international HIV/AIDS efforts in 1988, Bror Rexed, former General-Director of the National Board of Health and Welfare, highlighted the need for credibility, stating that:

But the experience of international cooperation must also be brought back to national work. Sweden becomes socially and scientifically credible first when one manages to prevent a widespread spread of HIV and AIDS in our own country [33].

In international work, the Swedish government and researchers took an early interest in HIV/AIDS, hosting the IV International AIDS Conference in Stockholm, sponsored by the Swedish Ministry of Health and Social Welfare, National Bacteriological Laboratory, the Karolinksa Institutet and the World Health Organization [81]. Sweden was also one of the largest early donors to the international AIDS response, including the WHO’s Global Programme on AIDS (GPA) [33, 82]. According to a 1988 memo regarding Sweden’s contribution to the GPA:

SIDA’s involvement with AIDS in the Third World has become a political issue in the campaign being waged for Sweden’s national elections this fall. Most of the political parties appear to be engaged in an attempt to outbid one another in terms of the financial contributions they would have SIDA commit [83].

This competition between political parties could be interpreted as an attempt to maintain Sverigebilden. That is, as Bergman Rosamond (2014) has argued, generous provisions of overseas development aid tied to ‘Sweden’s self-narrative as a provider of welfare at home and abroad,’ and thus it is difficult for any of the political parties to reduce financial commitments to aid [84].

Also during this time, Sweden’s entry in the EU in 1995 affected its domestic and global health approaches. Although Sweden was allowed to maintain its retail monopoly on the sale of alcohol, it was forced to liberalise other aspects, such as the production and wholesale [85]. More broadly, EU membership meant the Sweden could no longer be as independent in foreign policy and began to practice what Dahl has called a quieter diplomacy [86] In terms of global health and development Sweden began to channel action and funding through the EU (although this is only a minor percentage of total Swedish aid), and it can hypothesized that Sweden, in contrast to Norway, cannot speak as freely and act as independently as in the past because it must go along with the other EU Member States. At the same time, Sweden has used the EU as a platform to push for ‘poverty reduction, gender equality, environment, democratic development and human rights.’ [76]

While the late 1980s and 1990s presented challenges, this period also saw an increase in global attention to issues of women’s rights and to sexuality, and the solidification of Sweden’s approach to women’s rights – that gender equality is not possible without SRHR, and the creation of the term SRHR itself [59]. This was the era of the ‘big conferences’, such as the International Conference on Population and Development (ICPD) in Cairo (1994) and the Fourth World Conference on Women: Action for Equality, Development and Peace in Beijing (1995), which saw a further broadening of the narrowly-focussed family planning agenda to focus on human rights and wider social determinants of health [59]. More broadly, this was also the era in which gender equality began to feature explicitly as a part of *Sverigebilden*, in part due to a focus on gender equality in both domestic and foreign policy at the time [87, 88].

The late 1980s and early 1990s also saw the establishment of international health research groups within Sweden, such as the Umeå University Department of Epidemiology and Global Health and the Department of International Health Care Research (IHCAR) at Karolinska Institutet, the latter of which was characterised by close collaboration with Sida. This era also saw the establishment of the Alliance for Health Policy and Systems and Research at the WHO, with the support of the Swedish government and individual Swedish experts, such as Göran Tomson [89–91]. Additionally, the Swedish branch of Médecins Sans Frontières was founded in Sweden founded in the early 1990s by Johan von Schreeb, Anna Vejlens and Stefan Peterson.

### 2000–2010s: global health as a partnership Endeavour

The late 1990s and 2000s was characterised by a renaissance in global health, particularly with regard to financing and high-level political attention. The Millennium Development Goals were adopted in 2000 and within the health sector included a focus on reduction in child and maternal mortality, access to reproductive healthcare and reversing the spread of HIV/AIDS, tuberculosis and malaria. The MDGs were ‘instrumental in focussing global health resources in low and middle-income countries’ [92] and largely drove the global health agenda in the 2000s [93]. Global development assistance for health (DAH) increased substantially in the MDGs era, from US $10.8 billion in 2001 to $28.1 billion by 2012 (in 2010 US dollars). Swedish DAH also rose during this time, from 1.8 billion SEK in 2001 to about 4.3 billion SEK in 2014 [94, 95].

Taking over the helm of the WHO from Hiroshi Nakajima, Gro Harlem Brundtland responded to challenges of her predecessor by incorporating neoliberal language, such as ‘investing in health’ and by establishing initiatives such as the Commission on Macroeconomics and Health, a process to which several Swedes participated as members of constituent working groups [58]. The implementation of the MDGs was largely tied to public-private partnerships, themselves a resulted of the continued colonisation of neoliberal thinking within health.

As already noted, Swedish politics has gradually shifted to the right since the 1990s and the Alliance, a centre-right coalition, led the country from 2006 to 2014. Domestically, the Alliance also pushed for tax cuts, reductions in the welfare state and increased privatisation of social services, including health, although many of these policies in fact has originated with the Social Democrats in the late 1980s and early 1990s [84, 96].

In international work, the Alliance continued to promote the same norms and values around solidarity, justice and gender equality and promoted work that had been stressed by the previous Social Democratic governments, such as SRHR, including access to safe abortion [97]. They also continued the approach set out in the Policy for Global Development (2002) in which it was decided that that all government decisions should take into account poverty and equity [98], although the PGD was never quite fully realised, due to the ambiguities in formulation and the practical challenges of cross-sectoral action [99].

In contrast to the Social Democrats’ approach, the Alliance explicitly framed aid policy in pro-market and entrepreneurial terms, taking more of an explicit neoliberal stance on development [84]. Within this context, in which pro-market solutions were considered a way to address global health issues, public-private partnerships were seen as a clear way forward. While the Swedish government was initially critical of what they considered to be vertical approaches, under the Alliance government Sweden became one of the largest donors to emergent health partnerships, such as the Global Fund and Gavi [49]. Sweden was also keen to further the emergent interest with aid efficiency, with mixed results. For instance, in 2010 a serious set-back for Swedish aid came in the form of conflicts over results-based management reforms at Sida, along with significant overspending, which contributed to the firing of the Director-General, along with staff cuts of 25%. Additionally, around this same time there was a corruption scandal relating to Swedish aid in Zambia, which also highlighted concerns over aid effectiveness [21].

More positively, both domestically and internationally, antimicrobial resistance emerged as a profile issue for Sweden [91]. Already Sweden had a strong profile on pharmaceutical research and policy, as an early partner to the WHO on technical work on active substances and an early leader in pharmocoviligence systems which led to the establishment of the Uppsala Monitoring Centre to support the WHO Programme for International Drug Monitoring in 1978 [100]. In the 1980s, Sweden had eliminated the usage of antibiotics for growth promotion in livestock and had set up national campaigns around the appropriate use of antibiotics in the mid-1990s [101]. In the mid-1990s researchers such as Otto Cars, drew attention to the issue, initiating a national multidisciplinary group which later developed into the Strama network and, along with support from the Dag Hammarskjöld Foundation and researchers from IHCAR, set up ReAct, a global antibiotic resistance advocacy network [102]. During its 2009 presidency of the EU, Sweden drove the issue and also, with the UK, drove the Global Action Plan in 2015.

### 2015 – today: promoting sustainable development at home and abroad

In the 2019 Foreign Policy speech, Foreign Minister Margot Wallström set out Sweden’s approach to international development and health, noting the need for a ‘spirit of solidarity.’ She also reiterated the government’s commitment to pursuing a feminist foreign policy which in global health work means:

We will continue our extensive support to sexual and reproductive health and rights, for example by funding initiatives for legal abortions, contraceptives and sex education. We will work against trafficking in human beings, combat violence against women and share our experience of the Swedish Sexual Purchases Act [which criminalises the purchase, but not the sale, of sex] [103].

Sweden’s current approach to health and development is oriented around the Sustainable Development Goals (SDGs) (See Table 2). Developed as a successor to the MDG, which saw major strides in combating HIV, TB and malaria, the SDGs represent a substantial broadening of the development agenda to include all countries and to take an intersectoral focus. The SDGs, along with additional work carried out at the WHO and in other international fora demonstrates a broadened health agenda, addressing not only communicable diseases but also focussed on non-communicable diseases, including mental illness, substance abuse, road traffic accidents, SRHR target, pollution and universal health coverage.

**Table 2:** Current Policy Framework for Development Cooperation in Health [104] The aim of Swedish international development cooperation is to create preconditions for better living conditions for people living in poverty and under oppression. Good health development in the population is of fundamental importance for the development of society in general. Promoting health and preventing illness create conditions for long-term sustainability. Consequently, almost all the SDGs in the 2030 Agenda are important for people’s health and to the specific statement in SDG 3 on good health and well-being for all at all ages

Long-term policy directions include a focus on:	
• Effective national health systems;	
• Gender equality, including differences regarding health and access to health and medical care;	
• Child and maternity care;	
• All people’s right to health with a particular focus on sexual and reproductive health and rights.	
• Young people’s needs and the rights of LGBTQ people;	
• A long- term, rights-based and broad approach to combat the spread of HIV;	
• The importance of access to clean water, sanitation and hygiene, and sufficient, safe and nutritious food, as well as sustainable energy for health;	
• Non- communicable;	
• Antimicrobial resistance;	
• Implementation of the International Health Regulations (IHR 2005);	
• The link between health and environmental and climate challenges and between health and security	

*Adapted from the Government of Sweden’s Policy framework for Swedish development cooperation and humanitarian assistance. Government Communication 2016/17:60

Sweden took an early lead in the development of the SDGs. For example, the thematic consultation on health, was co-led by WHO and UNICEF, in collaboration with the Governments of Sweden and Botswana [99]. Within global health, Sweden’s action is targeted towards three main areas, all of which include a focus on SRHR: ‘Creating societal conditions for good and equitable health; health systems that are effective, sustainable and resilient; and improved preparedness and capacity to detect and manage outbreaks of diseases and other health threats.’ [105] Specifically, the formal goal of Sweden’s development cooperation for health is to promote equitable health, with priorities around sexual and reproductive health and rights – particularly within the context of Universal Health Coverage –, child and maternal health and health systems strengthening [106]. Two ways in which the Swedish government views itself as active in global health is through its role as a major donor to many multilateral organisations and also as an active member of the boards of alliances such as the Global Fund and Gavi [106].

Fulfilling the SDGs, at home and abroad, requires both research and credibility. One emergent initiative is the Swedish Institute for Global Health Transformation (SIGHT), which was launched in 2017 with the aim of invigorating interdisciplinary research and linking research, education and policy work [107]. Another specific example comes from the West Africa Ebola crisis (2014–2016), in which Swedes made a significant contribution, due in large part to the advocacy of individuals such as Hans Rosling and Johan von Screeb [108]. For instance, Lindstrand et al. describe how Hans Rosling encouraged quick action on Ebola:

“He held an epic speech at the Swedish Society of Medicine (Läkaresällskapet) about the importance of acting fast on the Ebola epidemic, which meant that all 400 participants stood up and were ready to leave the next day to stop the epidemic. Hans was central to mobilizing Swedish efforts to stop the epidemic and made sure that a course on how to work with Ebola infections, was arranged by the knowledge center for disaster medicine at KI [in only nine days] – the fastest arranged course in KI’s history.” [108]

Sweden’s long-standing approach to gender equality was further bolstered by the declaration in 2014 of a Feminist Foreign Policy which manifests itself in an explicit focus on gender equality and in specific policies, such as the Swedish Government’s response with other like-minded countries to fill the funding gap created by the United States’ reinstatement of the Mexico City Policy [109].

Finally, the 2010s has been characterized by a further reiteration of the Swedish Middle Way in Sweden’s approach to global health and SDGs more broadly. For instance, the government’s accounting of global health work in the context of Agenda 2030 states that:

“Business has an important role in global health work, both by driving the development and the dissemination of new technology and innovations ... There are, however, commercial forces that do not favour a positive development of health that are also important to pay attention to and counter [105].

This type of discourse is found also in Sweden’s strategies for cooperation with the WHO in which language around results-management and effectiveness are tied to rights and gender-equality perspectives [110, 111]. Ideally, this suggests an interpretation in which health is an investment, but not a commodity.

However, while Sweden ranks as the top country on SDG attainment, the county also faces many challenges. One challenge relates to the support of research which can support policy-making. In many ways, global health research in Sweden remains based on the narrow definition of global health as something that happens in low and middle-income countries, and as something that is medical, rather than interdisciplinary [112, 113]. Additionally, while there is general consensus that SRHR and AMR should remain profile issues for Sweden, this narrow focus on SRHR and AMR may prevent researchers from engaging in other key areas. As Sundewall et al. have noted, in the Swedish context ‘other important areas, such as health systems’ strengthening, communicable and non-communicable disease and prevention of chronic disease do not receive the same emphasis.’ [114] Additionally, Swedish researchers, like those in many other countries, often face such insecure work contracts, poor working environments and a lack of time and resources for research, with some commentators going so far as to denounce ‘cronyism and academic inbreeding’ at Swedish institutions [115, 116].

Other challenges relate to the sustainability of the Swedish Middle Way, such as cuts in the welfare sector, increasing health inequities, and the challenges of integrating vulnerable groups into society and the labour market, along with an increasing privatization of healthcare [117, 118]. Domestic scandals, such as the construction of the New Karolinska University Hospital via public-private partnership model, have drawn attention to healthcare organisation in Sweden. The hospital, which was meant to demonstrate an ultramodern, new way of organising care has been beset by rising costs, poor quality care and even accusations of corruption, most notably in the payment of external consultants [119, 120]. In addition, the intake of nearly half a million asylum seekers between 2010 and 2018 has also impacted the aid budget, in that some aid funding was shifted to support asylum seekers in Sweden [121]. More broadly, it has contributed to shifts in rhetoric around migration, with increased anti-immigrant sentiment in popular discourse. Finally, globally we have seen a plateauing health aid, which has further impacted the Swedish approach to aid [114]. These challenges present a threat to Swedish credibility in global health, which is arguably a precondition for *Sverigebilden*.

## Discussion

What it means to be ‘active’ or a ‘leader’ in global health is largely undefined. Research from other countries has identified the importance of being a large donor, hosting international meetings, engaging in various high-level panels, introducing World Health Assembly resolutions, supporting research and highlighting global health in non-health fora, such as the G7, G20 or UN Security Council; it also noted the importance of key individuals [14, 18]. Writing specifically of Sweden in the Lancet in 2017, chief editor Richard Horton wrote that the country had been a global health leader in the 1970s and 1980s, but that ‘for reasons unexplained and unexamined, Sweden slowly disengaged from the global health community.’ [15]

However, the article also finds no strong evidence that Sweden’s engagement in global health has decreased. Certainly, Sweden in the 1990s faced challenges, not least due to a recession; this also led Sida to re-orient its assistance to be more focusses on results and effectiveness [122]. There also there may be a visibility aspect. After joining the EU in 1995, Sweden aligns itself with statements of the EU in the World Health Assembly and other multilateral arenas. At the same time, by joining the EU, Sweden was forced to liberalise some of its policies, notably around alcohol, which impact public health. Unlike other major donors, Sweden is not a member of the G7 or an individual member of the G20 so these do not provide fora to exercise leadership. Individual Swedes have had high level position in international organisations, but throughout the past 70 years, there has been a sense that Swedes are under-represented in international organisations [4, 33, 123]. The challenges of reforms at Sida in the late 2000s/2010s and difficulties faced by researchers in Sweden may also have negatively affected the country’s engagement in global health, but Sweden remains an active country, particularly within the multilateral sphere. Indeed, this is seen both through significant financial commitments and engagement on the boards of international organisations. Sweden’s engagement has also become increasingly visible in sexual and reproductive health and rights: as SRHR language is regularly contested in multilateral negotiations, there is a view that Sweden’s role is increasingly important.

Taking a long view on global health engagement provides a context and explanation for how, over the course of a century and a half, one of the poorest countries in Europe became a significant player in global health. Not being an international colonial power put Sweden on a different trajectory, as did neutrality during WWI and WWII and non-alignment during the Cold War. A long period of peace and prosperity also created the domestic preconditions for welfare state development. This early development of the welfare state led to belief in the power of research to solve social ills, and we see a long tradition of research supporting both national and international policy, for instance the International Code of Marketing of Breastmilk Substitutes.

While Swedish aid has always been concerned about effectiveness, since the 1990s, there has been increasing focus on cost-effectiveness in line with neoliberal thinking. In large part, we see a ‘Middle Way’ interpretation which sees cost-effectiveness as a way to improve equity and promote rights. The stability of the government also contributes to Swedish credibility. Although Swedish politics were dominated by the Social Democrats in the twentieth Century, their positions were often not notably different than those of the other parties. Today the main political parties differ on the extent to which market-based solutions should play a role in development aid and global health, but the broad ideals of equity, rights and gender equality are largely shared values. This is also about cultivating *Sverigebilden*, which is based on the values of equal worth, dignity, respect, and collaboration. More broadly, in the Swedish context, this is underpinned in part by good will and a sense of responsibility and solidarity. This is the idea that ‘we have it good’ in Sweden and thus have a duty to help other countries. At the same time, there is also an aspect of self-interest: Sweden, as a small country, sees itself as dependent on a stable world.

Moving forward, Sweden faces two key interrelated challenges in regard to global health engagement. The first concerns research. If, global health, is to be understood as ‘those issues that transcend national boundaries and governments and call for actions on the global forced that determine the health of people [29], then Swedish approach to global health needs to be adjusted. There is need to embrace a wider conceptualisation of global health research that supports interdisciplinary collaborations to address transnational challenges. There is also a need to promote research on SRHR and AMR while also investing in other priorities found in the SDG framework, and to see climate change and sustainability as a part of health, not as a competitor for research funding.

Secondly, part of Sweden’s ability to be influential is based on the credibility of the Swedish model, the idea that innovation and wealth generation can be balanced with a comprehensive welfare state, and that this is dependent on accountable and well-functioning institutions. In international engagement this has meant being a trustworthy partner, particularly in the context of decolonialisation and as an activist donor. Credibility is also about supporting research and policy; for instance, Sweden’s role as a driver of SRHR and AMR is based on decades-long research and policy experience. There is a ‘co-constitutive relationship between states’ provisions of overseas development assistance and their domestic welfare commitments.’ [84] In many ways, both credibility and *Sverigebilden* are about the alignment of foreign and domestic policy.

## Conclusions

While an overview cannot capture the complexity or nuance of Sweden’s global health engagement, this article has highlighted a few of the episodes, debates and individuals that have contributed to the norms, values and traditions in Sweden’s approach to global health. This article contributes to an emerging literature on national contributions to global health and provides the first overview of a country which is often neglected in literature on global health governance and diplomacy. The article is also notable because it builds upon a number of Swedish-language sources that would otherwise be unavailable to an international audience.

The commitment to global health and development as a part of *Sverigebilden*, or the view of Sweden in the world. *Sverigebilden* vis-a-vis global health is characterised by norms and values such as human rights, solidarity, equity and gender quality. It is also characterized by a commitment to profile issues such as AMR and SRHR. These norms and values, in turn, were developed through social and political transformations in the domestic sphere. *Sverigebilden* is also dependent on credibility: Sweden is able to exercise influence because of a successful welfare model and strong research traditions.

Yet, *Sverigebilden* is a paradoxical concept. Writing of Swedish politics more broadly, Jerzierska & Towns (2018) have argued the dominant narrative of a progressive Sweden can hinder calls for domestic reform, particularly with regard to gender equality. In part, the fact the Sweden is starting from such a high level of health attainment means that a decline in health outcomes or living standards may be ignored because Sweden still performs well compared to other countries. It also means that inconsistencies in policy may be glossed over because, on balance, Swedish policies are ground in the principles of solidarity, equity, gender equality and cost-effectiveness. Sweden’s engagement and credibility in global health, while grounded in a strong historical basis, must be continuously re-negotiated and strengthened, via the relentless work of civil society, policy-makers, civil servants and voters.

## Data Availability

The manuscript is based on material available online or found in print in Swedish libraries, the latter of which can be found in the Swedish National Libraries database. Nordic newspaper sources can be found via the Retriever database. Other print material is physically located in the WHO archives or the Swedish National Archives. The interview material is not publicly available in order to protect individual privacy.
